# A national study on the physical and mental health of intersex adults in the U.S.

**DOI:** 10.1371/journal.pone.0240088

**Published:** 2020-10-09

**Authors:** Amy Rosenwohl-Mack, Suegee Tamar-Mattis, Arlene B. Baratz, Katharine B. Dalke, Alesdair Ittelson, Kimberly Zieselman, Jason D. Flatt

**Affiliations:** 1 Institute for Health and Aging, School of Nursing, University of California San Francisco, San Francisco, California, United States of America; 2 interACT: Advocates for Intersex Youth, Sudbury, Massachusetts, United States of America; 3 Androgen Insensitivity Syndrome-Differences of Sex Development Support Group, Duncan, Oklahoma, United States of America; 4 Department of Psychiatry, Penn State Milton S. Hershey Medical Center, Penn State College of Medicine, Hershey, Philadelphia, United States of America; 5 School of Public Health, Social and Behavioral Program, University of Nevada Las Vegas, Las Vegas, Nevada, United States of America; Universitat de Valencia, SPAIN

## Abstract

**Objectives:**

To describe the health of intersex adults (people with differences of sex development) in the U.S. using community-based research methods.

**Methods:**

In July–September 2018, we conducted a national health study of intersex adults aged 18 and older in the U.S., using a survey hosted on Qualtrics. The study describes the physical and mental health experiences of intersex adults, including differences by age (18 to 39 vs. 40 and older). Questions were derived from national (Behavioral Risk Factor Surveillance System) and intersex-related health studies.

**Results:**

A non-probability sample of 198 intersex adults completed the survey over three months. Over 43% of participants rated their physical health as fair/poor and 53% reported fair/poor mental health. Prevalent health diagnoses included depression, anxiety, arthritis, and hypertension, with significant differences by age. Nearly a third reported difficulty with everyday tasks and over half reported serious difficulties with cognitive tasks.

**Conclusions:**

To our knowledge, this is the first national study of intersex adults in the U.S. Greater understanding of intersex health over the life course is essential. Findings highlight the need for longitudinal studies and further examination of potential health disparities experienced by intersex populations.

## Introduction

Intersex variations, also known as differences of sex development (DSD), encompass a diverse set of congenital differences relating to gonads, chromosomes, and genitals that fall outside typical binary notions of male and female sex [1]. These may be identified in utero, at birth, in childhood, or in adulthood, and some may remain undiagnosed. It is challenging to estimate the prevalence of intersex conditions as no existing population studies include questions about intersex diagnoses, experts disagree on what conditions fall under the intersex category, and feelings of shame and stigma may limit disclosure by individuals [2]. Awareness and action on health disparities affecting lesbian, gay, bisexual, trans, and queer (LGBTQ) communities continue to increase [3], but intersex individuals, whether or not they identify as part of the LGBTQI umbrella, are often an invisible and forgotten group. The National Institutes of Health (NIH) recognizes intersex people as a sexual and gender minority (SGM) population affected by health disparities, highlighting “DSD populations” as a priority area for research [4].

Since the 1950s, medical care for intersex people has centered around surgical interventions in infancy that place individuals in binary sex categories without their consent, designed to “fix” ambiguities [5]. These non-consensual interventions can be irreversible and associated with long-term health challenges [6, 7]. It is clear that health care professionals, parents and caregivers, and intersex people differ in their preferences and priorities for care, with one study finding that pediatric urologists expressed particular concerns about difficulties with physical functioning but fewer concerns about gender-related issues compared to pediatric endocrinologists and advocates [8]. Following extensive advocacy by intersex people, several countries, international bodies, and American medical groups, including Human Rights Watch, the World Health Organization, and Amnesty International, have recommended delaying surgeries so that intersex people themselves can make informed decisions about what is best for them [7, 9–13]. Alongside this, a new community-centered research literature is beginning to emerge, highlighting intersex voices and experiences [14].

The focus on surgical intervention in childhood and infancy has contributed to a lack of understanding of the health needs of intersex adults over the life course. There are typically no dedicated services or plans of care for intersex adults, although it is clear that health needs relating to intersex conditions themselves as well as the effects of hormones and surgical interventions may continue throughout life [1]. Intersex adults may also face barriers to high-quality health care, including stigma and lack of health provider knowledge [7].

Most of the existing studies exploring the health of intersex adults were conducted in Europe through the dsd-LIFE research project [15]. Participants reported good health overall, but they experienced significantly higher rates of health problems compared to controls and were significantly more likely to report physical health limitations. Although this study has important implications, interventions and practices relating to intersex health may vary widely across countries, so these findings may not be applicable to the U.S. context. Relatively low rates of health insurance coverage in the U.S. may also affect health outcomes [16]. Unfortunately, research on the health of intersex adults in the U.S. is very limited [17].

The aim of this study was to explore the physical and mental health of intersex adults in the U.S., through a community-based partnership with community advocates and health care professionals. In this manuscript, we describe the design and recruitment of the first U.S. intersex adult health study, including community partner involvement in survey development and outreach and descriptive findings on the demographic and health characteristics of the study sample. We also explore differences in demographics, physical and mental health conditions, and functional impairment by age (18 to 39 vs. 40 and older).

## Methods

### Design

The target population for this study was intersex adults aged 18 and older currently living in the U.S. This underserved and marginalized SGM population is often hard to recruit due to past experiences of trauma, exploitation, and stigmatization in research and clinical settings. We recruited a non-probability sample using community-engaged research approaches to maximize participation. The survey was distributed via Qualtrics, a secure web-based survey platform, from July to September 2018. Ethics approval was granted by the University of California, San Francisco Institutional Review Board.

In developing the survey and recruitment plan, we partnered with community members including intersex adults, family members of intersex people, and physicians and policy advocates involved in intersex care and advocacy. Many of the existing studies on procedures and outcomes for intersex children are conducted and published by surgeons, so their perspective is already well represented. In this study, our focus was on the lived experiences of those who are personally affected. Our community partners were fully involved at each stage of the survey development and recruitment, from initial decisions about topic areas to testing and refining the survey tool. The content and structure of the survey were developed iteratively with our community partners over four months. Prior to data collection, the survey tool was pilot tested with community partners to evaluate usability, technical functionality, and acceptability of wording. The survey was comprised of 84 questions with adaptive questioning and skip patterns to reduce burden.

The survey covered four key areas: 1) demographics; 2) intersex-related health diagnoses; 3) physical health; and 4) mental health. Whenever possible, questions were adapted from existing validated measures, although previous research with and validated health measures for this population are extremely limited. When no previous measures were available, we developed tailored questions through community partner review and pilot testing.

### Measures

The majority of health-related questions were derived from the Behavioral Risk Factor Surveillance System (BRFSS) [18] and existing studies with intersex adults and sexual and gender minority populations (see S1 and S2 Files) [6, 19–22].

#### Demographic variables

Demographic data included age, sex assigned at birth, gender identity, sexual orientation, race/ethnicity, education level (no schooling to professional degree), place of birth (U.S. or elsewhere), annual household income, financial difficulties, employment status, relationship status, health care coverage, and participation in U.S. Armed Services [18, 22].

#### Intersex-related health diagnoses

Questions on diagnoses of intersex-related variations were based on previous research [2, 23] and expertise of our community partners and research team.

#### Physical, psychological, and functional health

Questions included self-rated physical, functional, and mental health, as well as self-reported health conditions. Questions on health conditions included ever being told you have heart disease, cancers, stroke, osteoporosis, arthritis, and kidney disease. We also asked participants about sensory problems and daily functioning. For mental health, questions included ever being told you have a diagnosis of depression, anxiety, and post-traumatic stress disorder (PTSD), as well as the 4-item Center for Epidemiological Studies Depression (CES-D) symptoms index, with scores ranging from 0–12 and a cutoff score ≥4 defined as elevated depressive symptoms [24, 25]. The CES-D is intended to be used as a screening tool for depressive symptoms over the past week and is not diagnostic in itself.

### Survey administration

The survey began with a study explanation and online consent form, with information on the estimated time to complete, principal investigator, data security and confidentiality protections, and purpose of the study. Those who provided informed consent and met eligibility criteria (aged 18 and older, previously diagnosed with an intersex variation, currently living in the U.S.) were invited to participate in the study. As an incentive for survey completion, participants had the option of providing an email address to enter a drawing for one of 50 $10 gift cards. Email addresses were kept separate from survey responses to ensure confidentiality. The survey was launched at the Androgen Insensitivity Syndrome-Differences of Sex Development (AIS-DSD) Support Group conference in Chicago, IL in July 2018, followed by extensive social media recruitment led by our community partners through September 2018. Recruitment included online flyers via social media, word of mouth, and targeted outreach to online support groups (see S3 File).

### Data analysis

Utilizing a structured protocol from the Checklist for Reporting Results of Internet E-Surveys (CHERRIES) [26], all survey responses were checked for eligibility based on inclusion criteria. Entries were examined carefully by the research team by comparing IP addresses, duplicate responses, incomplete responses, and missing responses. Given that two or more eligible participants may have shared the same household or computer, multiple responses from the same IP address were not deleted if responses to demographic and health questions were distinctly different. If multiple responses were similar but one response was incomplete, only the most complete response was kept.

Descriptive statistics were calculated to describe the health of participants, stratified by age (18 to 39 vs. 40 and older). The cut point was based on the age distribution of our sample, with a mean of 37 and a median of 34 years, as well as previous research using BRFSS identifying 40 years as an appropriate lower bound for middle age [27]. Nine participants did not disclose their age and are therefore not included in subgroup analyses, although their data were retained for statistics on the full sample. Since the number of participants who responded to each question varied (participants could skip sensitive questions), we highlight in tables the number who selected each response as well as the percentage. This approach was chosen to maximize inclusion of participant data, acknowledging that participants may prefer not to respond due to past trauma and/or discrimination. The Pearson χ^2^ test of independence was used to test whether health and functional outcomes differed by age group, with Fisher’s exact test used where cells had low expected frequencies (<5). We used logistic regression models to explore associations between demographics and measures of health status and outcomes (self-reported depression diagnosis, self-reported anxiety diagnosis, disability, and self-reported chronic disease). Independent variables were selected based on prior research and were entered into each model simultaneously. Odds ratios and 95% confidence intervals are reported. All statistical analyses were conducted using SPSS (Statistical Package for Social Sciences) version 24.0.

## Results

Two hundred and fifty-nine participants attempted to complete the survey between July and September 2018. Of these participants, one did not consent, three did not answer the first screening question (current U.S. residence), 18 did not live in the U.S., eight did not answer the second screening question (intersex diagnosis), and 10 had not been diagnosed with an intersex variation. A total of 40 participants did not complete the eligibility questions for the above reasons, and these entries were deleted. After reviewing all remaining cases with identical IP addresses, five were confirmed as duplicate entries and deleted. Finally, 16 of the remaining participants did not answer beyond the eligibility section, or only answered one or two questions throughout the survey. These entries were also deleted. The total number of deleted cases was 61, resulting in a final analytic sample of 198 responses. Of these, 179 (90.4%) completed the entire survey.

### Demographics

Table 1 describes the demographics of the study sample, stratified by age group. The mean age of intersex adults in this study was 37.6 years (SD = 14.3), with a range of 18 to 78 years. Overall, 2.2% of intersex adults in this study identified as American Indian/Alaska Native, 2.8% Asian, 1.7% Black/African American, and 72.8% white, with 6.7% endorsing another race not listed and 13.9% endorsing more than one race. Nearly 14% identified as Latinx/Hispanic, and 88.3% reported that they were born in the U.S. With regards to sex assigned at birth, the majority (66.3%) were assigned female at birth; however, 3.7% did not know their sex assigned at birth. When asked about current gender identity, with multiple selections permitted, the majority (63.4%) identified as intersex, with 44.0% identifying as women, 7.9% as trans women, 12.0% as men, and 7.9% as trans men. Over 25% of participants reported identifying as non-binary, 9.4% genderqueer, and 17.8% another gender identity (note: multiple selections permitted). For sexual orientation, the most commonly reported identities included queer (28.0%), bisexual (26.5%), and straight/heterosexual (24.9%). For relationship status, nearly half were currently single, separated, or divorced, 37.7% were married or partnered, 1.6% were widowed, and 12.6% reported another relationship status.

**Table 1:** Demographic characteristics.

	Overall	Younger group (age 18 to 39)	Older group (age 40+)
	N = 198	n = 118	n = 71
	*n (%)*	*n (%)*	*n (%)*
Age in years *Mean (sd)*	37.56 (14.3)	28.05 (6.1)	53.35 (9.2)
*Range*	18–78	18–39	40–78
Race			
American Indian/Alaska Native	4 (2.2%)	0 (0.0%)	4 (5.9%)
Asian	5 (2.8%)	4 (3.7%)	1 (1.5%)
Black or African American	3 (1.7%)	3 (2.8%)	0 (0.0%)
Native Hawaiian/Other Pacific Islander	0 (0%)	0 (0%)	0 (0%)
White	131 (72.8%)	80 (73.4%)	50 (73.5%)
Another race	12 (6.7%)	8 (7.3%)	4 (5.9%)
Two or more races	25 (13.9%)	14 (12.8%)	9 (13.2%)
Ethnicity			
Latino/a/x or Hispanic	25 (13.6%)	17 (15.3%)	7 (10.1%)
Born in the U.S.	166 (88.3%)	96 (83.5%)	67 (97.1%)
Sex assigned at birth			
Female	126 (66.3%)	82 (71.9%)	42 (59.2%)
Male	57 (30.0%)	27 (23.7%)	27 (38.0%)
Don’t know/Not sure	7 (3.7%)	5 (4.4%)	2 (2.8%)
Current gender identity^a^			
Intersex	121 (63.4%)	80 (69.0%)	39 (54.9%)
Trans woman	15 (7.9%)	9 (7.8%)	5 (7.0%)
Woman	84 (44.0%)	47 (40.5%)	35 (49.3%)
Trans man	15 (7.9%)	11 (9.5%)	4 (5.6%)
Man	23 (12.0%)	12 (10.3%)	10 (14.1%)
Genderqueer	18 (9.4%)	16 (13.8%)	2 (2.8%)
Non-binary	52 (27.2%)	40 (34.5%)	12 (16.9%)
Another gender identity	34 (17.8%)	18 (15.5%)	16 (22.5%)
Sexual orientation^a^			
Asexual	32 (16.9%)	26 (22.4%)	5 (7.2%)
Bisexual	50 (26.5%)	35 (30.2%)	15 (21.7%)
Gay	16 (8.5%)	12 (10.3%)	4 (5.8%)
Lesbian	29 (15.3%)	18 (15.5%)	10 (14.5%)
Pansexual	42 (22.2%)	33 (28.4%)	9 (13.0%)
Queer	53 (28.0%)	42 (36.2%)	10 (14.5%)
Questioning	13 (6.9%)	10 (8.6%)	3 (4.3%)
Same-gender loving	14 (7.4%)	10 (8.6%)	4 (5.8%)
Straight/heterosexual	47 (24.9%)	19 (16.4%)	26 (37.7%)
Another sexual orientation	16 (8.5%)	7 (6.0%)	9 (4.9%)
Relationship status			
Single, separated, or divorced	92 (48.2%)	73 (62.4)	18 (25.7%)
Married or partnered	72 (37.7%)	27 (23.1%)	42 (60.0%)
Widowed	3 (1.6%)	0 (0.0%)	3 (4.3%)
Another relationship status	24 (12.6%)	17 (14.5%)	7 (10.0%)
Employment status			
Currently working	110 (58.5%)	71 (62.8%)	36 (50.7%)
Disabled or unable to work	29 (15.4%)	16 (14.2%)	13 (18.3%)
Homemaker	7 (3.7%)	2 (1.8%)	5 (7.0%)
Retired	15 (8.0%)	0 (0.0%)	15 (21.1%)
Unemployed or laid off and looking for work	20 (10.6%)	17 (15.0%)	2 (2.8%)
Another	25 (13.3%)	16 (14.2%)	9 (12.7%)
Income			
$0–20,000	44 (25.6%)	31 (30.4%)	12 (17.9%)
$20,001–30,000	20 (11.6%)	17 (16.7%)	3 (4.5%)
$30,001+	108 (62.8%)	54 (52.9%)	52 (77.6%)
Worry about meeting expenses?			
Never	40 (21.2%)	21 (18.3%)	18 (25.7%)
Sometimes	58 (30.7%)	30 (26.1%)	27 (38.6%)
Often	42 (22.2%)	30 (26.1%)	11 (15.7%)
Always	49 (25.9%)	34 (29.6%)	14 (20.0%)
Education			
High school graduate or below	21 (10.9%)	19 (16.2%)	2 (2.8%)
Some college, technical training, or 2-year college degree	54 (28.1%)	35 (29.9%)	18 (25.4%)
4-year college degree	69 (35.9%)	44 (37.6%)	23 (32.4%)
Master’s degree or higher	48 (25.0%)	19 (16.2%)	28 (39.4%)
Currently or previously in the US Armed Services	13 (6.7%)	3 (2.5%)	10 (14.1%)

^a^ Participants could select multiple responses

In terms of socioeconomic status, nearly 60% were currently working, with 15.4% reporting being disabled or unable to work and 10.6% unemployed. About four percent reported being homemakers, and 8.0% were retired, with 13.3% reporting another employment situation. Veterans and current members of the U.S. Armed Services made up 6.7% of the total sample. Over 50% reported a household income of $40,000 or less, with over 1 in 4 reporting annual household income of ≤$20,000. In addition, 48.1% reported frequently worrying about not meeting expenses with their current income. For educational attainment, 35.9% had completed a four-year college degree and one in four had a graduate degree.

### Intersex variations

The most frequently reported intersex diagnoses were complete AIS (19.1%), partial AIS (18.6%), micropenis (14.9%), clitoromegaly (14.9%), ovo-testes (12.9%), and hypospadias (11.9%), with over 30 different diagnoses represented in the sample (see Table 2). Over 10% of participants reported not knowing what intersex variation they had. In addition, nearly 50% of participants reported two or more intersex diagnoses. The average age at which participants reported first finding out about their diagnosis was 20.6 (SD = 12.3), and those in the younger age group found out at a significantly younger age (16.8 vs. 26.3 years, p<0.005).

**Table 2:** Intersex diagnoses.

	Whole sample	Younger group (age 18 to 39)	Older group (age 40+)	*p* value
	*n (%)*	*n (%)*	*n (%)*	
Intersex diagnoses^a^				
Complete AIS	37 (19.1%)	24 (20.3%)	13 (18.3%)	
Partial AIS	36 (18.6%)	21 (17.8%)	15 (21.1%)	
Clitoromegaly	29 (14.9%)	19 (16.1%)	10 (14.1%)	
Micropenis	29 (14.9%)	13 (11.0%)	16 (22.5%)	
Ovo-testes	25 (12.9%)	16 (13.6%)	8 (11.3%)	
Hypospadias	23 (11.9%)	12 (10.2%)	10 (14.1%)	
Gonadal dysgenesis	17 (8.8%)	10 (8.5%)	7 (9.9%)	
Mosaicism	16 (8.2%)	8 (6.8%)	7 (9.9%)	
Klinefelter syndrome	13 (6.7%)	5 (4.2%)	8 (11.3%)	
PCOS/hyperandrogenism	13 (6.7%)	11 (9.3%)	2 (2.8%)	
Swyer syndrome	13 (6.7%)	5 (4.2%)	8 (11.3%)	
Classic CAH	11 (5.7%)	9 (7.6%)	2 (2.8%)	
XXY/47	11 (5.7%)	2 (1.7%)	9 (12.7%)	
MRKH	10 (5.2%)	9 (7.6%)	1 (1.4%)	
Cryptorchidism	9 (4.6%)	4 (3.4%)	5 (7.0%)	
5-ARD	6 (3.1%)	5 (4.2%)	1 (1.4%)	
Persistent Mullerian duct syndrome	6 (3.1%)	1 (0.8%)	4 (5.6%)	
XY-XO Mosaics	4 (2.1%)	1 (0.8%)	3 (4.2%)	
17-Beta hydroxysteroid dehydrogenase deficiency	3 (1.5%)	1 (0.8%)	2 (2.8%)	
Bladder exstrophy	3 (1.5%)	1 (0.8%)	2 (2.8%)	
De la Chapelle (XX male) syndrome	3 (1.5%)	1 (0.8%)	1 (1.4%)	
Late onset CAH	3 (1.5%)	2 (1.7%)	0 (0.0%)	
Epispadias	2 (1.0%)	2 (1.7%)	0 (0.0%)	
Mullerian (Duct) aplasia	2 (1.0%)	2 (1.7%)	0 (0.0%)	
Progestin induced virilization	2 (1.0%)	0 (0.0%)	2 (2.8%)	
XY-Turner syndrome	2 (1.0%)	1 (0.8%)	1 (1.4%)	
Aphallia	1 (0.5%)	1 (0.8%)	0 (0.0%)	
Fraser syndrome	1 (0.5%)	1 (0.8%)	0 (0.0%)	
Jacobs/XYY syndrome	1 (0.5%)	1 (0.8%)	0 (0.0%)	
Kallmann syndrome	1 (0.5%)	0 (0.0%)	1 (1.4%)	
Leydig cell hypoplasia	1 (0.5%)	1 (0.8%)	0 (0.0%)	
Turner syndrome (TS, one X chromosome)	1 (0.5%)	1 (0.8%)	0 (0.0%)	
Triple-X syndrome (XXX)	1 (0.5%)	1 (0.8%)	0 (0.0%)	
Unknown	21 (10.8%)	14 (11.9%)	6 (8.5%)	
Another variation	19 (9.8%)	9 (7.6%)	10 (14.1%)	
Age found out about intersex diagnosis *mean (sd)*	20.58 (12.3)	16.83 (6.8)	26.33 (16.1)	*p*<0.005

^a^ Participants could select multiple responses

### Physical health

We examined chronic conditions and overall physical, functional, and mental health (Tables 3 and 4). More than 1 in 3 participants reported their physical health as fair/poor, which did not differ significantly by age group. The most commonly reported physical health diagnoses were asthma (27.3%), arthritis, gout, lupus, or fibromyalgia (27.3%), high blood pressure (24.7%), and osteoporosis (22.7%). Rates of physical health diagnoses were higher in the older age group, with significantly higher rates of high blood pressure (p<0.01), stroke (p = 0.01), arthritis (p<0.01), osteoporosis (p = 0.04), and diabetes (p<0.01).

**Table 3:** Physical health conditions and functional difficulties.

	Whole sample	Younger group (age 18 to 39)	Older group (age 40+)	*p* value
	*n (%)*	*n (%)*	*n (%)*	
General physical health:				0.450
Excellent	10 (5.1%)	6 (5.1%)	3 (4.2%)	
Very good	42 (21.3%)	26 (22.0%)	15 (21.1%)	
Good	60 (30.5%)	35 (29.7%)	22 (31.0%)	
Fair	55 (27.9%)	29 (24.6%)	24 (33.8%)	
Poor	30 (15.2%)	22 (18.6%)	7 (9.9%)	
Physical health diagnoses				
High blood pressure	49 (24.7%)	17 (14.4%)	28 (39.4%)	*p*<0.005
Heart attack/myocardial infarction	5 (2.5%)	3 (2.5%)	2 (2.8%)	1.000
Angina or coronary heart disease	7 (3.5%)	3 (2.5%)	3 (4.2%)	0.833
Stroke	6 (3.0%)	0 (0.0%)	5 (7.0%)	0.014
Asthma	54 (27.3%)	32 (27.1%)	21 (29.6%)	0.716
Skin cancer	11 (5.6%)	3 (2.5%)	7 (9.9%)	0.066
Other cancers	12 (6.1%)	5 (4.2%)	6 (8.5%)	0.380
COPD	8 (4.0%)	2 (1.7%)	6 (8.5%)	0.063
Arthritis, gout, lupus, fibromyalgia	54 (27.3%)	21 (17.8%)	30 (42.3%)	*p*<0.005
Osteoporosis	45 (22.7%)	21 (17.8%)	22 (31.0%)	0.036
Kidney disease	12 (6.1%)	5 (4.2%)	7 (9.9%)	0.220
Diabetes	23 (11.6%)	8 (6.8%)	15 (21.1%)	0.003
Deaf or serious difficulty hearing	18 (9.7%)	11 (10.1%)	6 (8.7%)	0.758
Blind or serious difficulty seeing	17 (8.7%)	10 (8.6%)	6 (8.5%)	0.968
Serious difficulty walking or climbing stairs	43 (22.8%)	23 (20.4%)	19 (27.5%)	0.265
Difficulty dressing or bathing	16 (8.3%)	13 (11.4%)	3 (4.3%)	0.096
Difficulty doing errands alone due to physical, mental, or emotional condition	59 (30.9%)	40 (35.1%)	17 (24.6%)	0.139
Serious difficulty concentrating, remembering, or making decisions	98 (56.6%)	71 (66.4%)	24 (40.7%)	0.001

**Table 4:** Mental health conditions.

	Whole sample	Younger group (age 18 to 39)	Older group (age 40+)	*p* value
	*n (%)*	*n (%)*	*n (%)*	
General mental health:				0.001
Excellent	7 (3.6%)	4 (3.4%)	2 (2.9%)	
Very good	37 (19.1%)	15 (12.8%)	22 (31.9%)	
Good	46 (23.7%)	24 (20.5%)	19 (27.5%)	
Fair	63 (32.5%)	41 (35.0%)	21 (30.4%)	
Poor	41 (21.1%)	33 (28.2%)	5 (7.2%)	
Mental health diagnoses				
Depressive disorder	121 (61.1%)	73 (61.9%)	45 (63.4%)	0.835
Anxiety disorder	124 (62.6%)	84 (71.2%)	36 (50.7%)	0.005
PTSD	81 (40.9%)	51 (43.2%)	27 (38.0%)	0.483
CES-D 4-item positive screen for depression (≥4)	119 (61.7%)	80 (68.4%)	35 (50.0%)	0.012
Ever attempted suicide				0.587
Never	27 (14.3%)	13 (11.4%)	12 (17.6%)	
It was just a brief passing thought	48 (25.4%)	27 (23.7%)	19 (27.9%)	
Had a plan at least once but did not try to do it	54 (28.6%)	34 (29.8%)	19 (27.9%)	
Attempted but did not want to die	19 (10.1%)	14 (12.3%)	5 (7.4%)	
Attempted and really hoped to die	41 (21.7%)	26 (22.8%)	13 (19.1%)	

### Sensory and functional issues

Among the entire sample, 9.7% reported being deaf or having serious difficulty hearing, and 8.7% reported being blind or having serious difficulty seeing; rates did not differ significantly by age group. In terms of functional status, 22.8% reported serious difficulty walking or climbing stairs, 8.3% difficulty with dressing or bathing, and almost one third difficulty doing errands alone due to their physical, mental, or emotional health. More than half reported serious difficulty concentrating, remembering, or making decisions (cognitive tasks). There were no significant differences by age group except for concentration, memory, and decision-making, where problems were more likely to be reported by younger participants compared to the older participants (66.4% vs. 40.7%, p = 0.001).

### Mental health

More than half of participants (53.6%) described their mental health as fair/poor. Self-reported mental health varied significantly by age group (p = 0.001); for example, 28.2% of the younger age group reported poor mental health compared to just 7.2% in the older age group. In terms of mental health diagnoses, 61.1% reported ever having been told they had a depressive disorder, 62.6% an anxiety disorder, and 40.9% PTSD. Rates of depression and PTSD did not differ significantly by age group, whereas a significantly higher proportion of the younger group reported a diagnosis of an anxiety disorder (71.2% vs. 50.7%, p<0.01). The prevalence of depression diagnosis was similar to the results of the CES-D 4-item scale, with 61.7% of the sample screening positive for clinically significant depressive symptoms in the past week (score of ≥ 4). A significantly higher percentage of the younger age group screened positive on the CES-D 4-item index compared to the older group. Almost a third of participants reported that they had previously attempted suicide; responses to this question did not differ significantly by age group.

### Multivariate analyses

The results of our logistic regression models (Table 5) indicate that being worried about money and reporting a diagnosis of anxiety were significant predictors of self-reported lifetime depression diagnosis, and younger age and reporting a diagnosis of depression were significant predictors of self-reported lifetime anxiety diagnosis. For disability, significant predictors were being a person of color, having worse self-reported physical health, and having worse self-reported mental health. Finally, reporting at least one chronic health problem was significantly associated with older age and worse self-reported physical health.

**Table 5:** Multivariate analyses.

*Outcome Predictor*	Depression	Anxiety	Disability	Chronic disease
*Age*	1.02 (0.98–1.07)	0.96 (0.93–1.00)*	0.99 (0.94–1.04)	1.10 (1.05–1.16)***
*Person of color*	1.92 (0.55–6.66)	0.50 (0.17–1.49)	12.22 (2.01–74.34)**	0.54 (0.18–1.64)
*Latinx*	2.62 (0.37–18.74)	1.75 (0.33–9.28)	3.52 (0.26–47.34)	0.38 (0.08–1.89)
*Worried about money*	9.44 (2.56–34.83)***	0.70 (0.21–2.31)	3.65 (0.82–16.32)	0.58 (0.15–2.28)
*Education*				
*High school or below*	*Reference*	*Reference*	*Reference*	*Reference*
*Some college*	1.07 (0.13–8.70)	4.11 (0.60–28.15)	0.50 (0.09–2.83)	0.79 (0.11–5.50)
*4 year college degree*	0.54 (0.07–4.37)	2.46 (0.36–16.72)	0.37 (0.07–1.90)	0.66 (0.09–4.76)
*Masters or higher*	1.33 (0.14–12.60)	2.12 (0.28–15.97)	*Omitted*	0.52 (0.06–4.40)
*Employed*	1.10 (0.40–2.99)	0.92 (0.37–2.29)	0.60 (0.15–2.36)	0.69 (0.24–1.99)
*Physical Health*				
*Good to excellent*	*Reference*	*Reference*	*Reference*	*Reference*
*Fair/poor*	2.76 (0.86–8.86)	1.50 (0.50–4.52)	27.2 (2.88–257.00)**	3.54 (1.13–11.06)*
*Mental Health*				
*Good to excellent*	*Reference*	*Reference*	*Reference*	*Reference*
*Fair/poor*	2.73 (0.86–8.70)	0.71 (0.24–2.10)	11.44 (2.59–50.59)***	0.61 (0.19–1.92)
*Disability*	0.55 (0.15–2.00)	1.93 (0.55–6.68)	-	3.14 (0.85–11.52)
*Chronic disease*	1.03 (0.33–3.26)	1.09 (0.39–3.07)	2.77 (0.66–11.59)	-
*Anxiety*	6.03 (2.21–16.42)***	-	2.45 (0.66–9.03)	0.96 (0.33–2.73)
*Depression*	-	5.96 (2.22–15.97)***	0.58 (0.14–2.39)	1.08 (0.36–3.27)

All statistics reported as odds ratios (95% CI)

* = p<0.05

** = p<0.01

*** = p<0.001

## Discussion

### Summary of findings

This is the first research study to our knowledge to explore the physical and mental health experiences of intersex adults in the U.S. We demonstrated the feasibility of using online survey methods to investigate the health of this population, as well as drawing on community partnerships to maximize participation and survey completion. Our participants represented a range of ages, gender identities, sexual orientations, and intersex diagnoses. Although this pilot study did not include a comparison group, questions were designed to enable us to compare our findings with larger-scale datasets, and we identified differences by age.

Our findings indicate several important strengths in terms of intersex health and wellbeing. The majority of intersex adults in this study reported good or better physical health, and almost half reported good or better mental health. In general, the prevalence of physical health conditions was low. However, we also identified several areas of significant concern, particularly in terms of function and mental health.

#### Physical and functional health

Our primary comparison is with national BRFSS data, which is weighted to reflect state and national population demographics. Our sample is clearly younger compared to the US population; 62% of our intersex survey respondents were aged 18–39, with only 4% aged 65 or above, whereas 62% of the national population is aged 18–64 [28]. The race/ethnicity of our sample is similar to BRFSS, except for underrepresentation of Black/African American (1.7% vs 12.8%) and overrepresentation of mixed race (13.9% vs. 1.6%) participants. Levels of employment, relationship status, and income are similar, although our sample includes more very low wage earners (under $15,000). Our sample also has more years of education compared to BRFSS.

Given the age differences between our sample and the US population sampled by BRFSS, we would expect to see better self-reported health in our study, since this typically declines with age and increases with higher income and education [29]. However, we found that intersex adults reported worse physical health when compared to national BRFSS data from 2017 [30], with over 43% of participants reporting fair/poor general physical health, compared to 17.7% in the general population. In contrast, our sample had lower or similar prevalence of most of the physical health conditions compared to BRFSS data. However, we noted higher prevalence of asthma (27.3% vs. 14.11%) among intersex adults compared to BRFSS, which is consistent with the younger age of our participants. Further investigation is warranted to explore age/cohort effects on self-reported health status as well as physical health diagnoses among intersex adults, particularly in light of the surprisingly poor self-reported health in this sample.

Functional status appeared to be more impaired in our sample compared to BRFSS, in spite of the younger age of our participants [31], with a higher prevalence of difficulties with concentrating (56.6% vs. 11.31%), walking or climbing stairs (22.8% vs. 14.1%), dressing or bathing (8.3% vs. 4.11%), and doing errands alone (30.9% vs. 7.24%). These findings may be reflective of the poor mental health status of our sample and/or undiagnosed physical health problems that affect function. Additional research is needed to examine the relationship between health and functional status among intersex adults of all ages.

U.S. data on intersex adult health is limited, but the European dsd-LIFE study provides large-scale comparison data [15]. dsd-LIFE recruited over 1000 adults with confirmed diagnoses falling under the DSD clinical umbrella from six European Union member states. The sample was similar to our sample in terms of age, with mean age at 32.4 years (SD = 13.6, range 16–75), but the European investigators used different recruitment and assessment strategies, primarily recruiting current and former patients of DSD clinical centers and confirming diagnoses and health status through chart review and physical examination. The distribution of intersex variations in their sample was also different, with higher representation of Turner and Klinefelter syndromes. In spite of these limitations, dsd-LIFE provides the most appropriate comparison data currently available on intersex health.

Overall, dsd-LIFE found that intersex individuals also experienced significantly higher prevalence of health problems (fair/poor self-rated health, several physical and mental health diagnoses) compared to controls. When comparing our study findings to dsd-LIFE, we find that U.S. intersex adults in our study had worse self-reported health as well as higher prevalence of all of the health conditions, including diabetes (11.6% vs. 4.1%), hypertension (24.7% vs. 11.0%), osteoporosis (22.7% vs. 10.7%), and joint problems (27.3% vs. 10.6%). This may reflect poorer health outcomes in general in the U.S. compared to European countries, as well as specific factors impacting the health of intersex adults in the U.S. and differences in study design.

#### Mental health

The responses to our survey questions on mental health reveal a concerning picture. In terms of diagnoses, we found high prevalence of self-reported lifetime anxiety disorders (62.6%), PTSD (40.9%), and depressive disorders (61.1%), as well as positive screening for current depressive symptoms (61.7%). We also found significantly higher prevalence of anxiety among younger participants, consistent with cohort trends in the general population [32]. In comparison, the prevalence of depressive disorder was more than three times lower (19%) in 2017 BRFSS [30]. The BRFSS question bank does not include CES-D screening, but there is a measure of self-assessed mental health over the past 30 days: “How many days was your mental health, which includes stress, depression, and problems with emotions, not good?” Reporting 14 or more days of “not good” mental health over the past 30 days is characterized by the CDC as Frequent Mental Distress (FMD), and the prevalence of FMD in the weighted 2017 BRFSS data was 13%.

Our study’s findings are comparable to the European dsd-LIFE research project, which reported significantly higher rates of psychiatric disorders evaluated through medical examination (including anxiety, depression, and schizophrenia, as well as eating disorders, autism, and attentional/behavioral disorders) among intersex participants compared to controls (p<0.0001) [15]. The researchers found that being older when diagnosed with an intersex condition predicted psychiatric disorder diagnosis. In another report on the dsd-LIFE data, the authors found that shame, stigma, low self-esteem, and low satisfaction with healthcare were associated with clinically significant symptoms of depression and anxiety [33]. Finally, a study on quality of life among dsd-LIFE participants found that good self-reported health status was the most important predictor of psychological quality of life; the authors concluded that providing good physical and mental health care to intersex people is likely to improve their quality of life [34]. In our intersex health study, we found that socio-demographic characteristics such as age and income inadequacy were significantly associated with mental health outcomes, similar to population trends [35]. However, it is important to note that we did not examine associations with any measures of shame, stigma, discrimination, or experiences with healthcare (such as surgical procedures or genital exams). Future research should consider these and other unique experiences of intersex adults in order to develop a more comprehensive and nuanced understanding of their health and wellbeing.

In our study, 31.8% of participants reported that they had previously attempted suicide. The dsd-LIFE study authors [15] found that 6.8% of participants had attempted suicide, which was significantly higher than the prevalence of 1.8% in their control group. An Australian online survey conducted with intersex people aged 16–87 found 19% had attempted suicide [36]. In the U.S., the lifetime prevalence of suicide attempts is 4.6% [37], and the prevalence in our intersex study is comparable to the lifetime suicide attempt rate for transgender people, which is around 41% [38]. According to the Williams Institute, higher rates of suicide attempts among transgender and gender non-conforming adults may be due to experiences of discrimination, violence, and refusal of medical treatment [38]. Similarly, the high rate of suicidality and poor mental health in our cohort may be due to adverse experiences including high rates of stigma and discrimination from society and poor treatment in healthcare settings. Further research is needed to identify risk factors for the elevated mental health problems and suicide attempts among U.S. intersex adults in our sample as well as prevention strategies.

### Limitations and strengths

The primary strength of this research study is that it contributes new knowledge on the health of intersex adults in the U.S. and provides points of comparison with existing U.S. samples and previous European studies. Using extensive online and in-person outreach coupled with community-engaged research approaches, we maximized participation and response/completion rates. This study was designed carefully through close collaboration with a group of community partners and expert health care providers, using questions from large-scale national studies to support comparison.

We acknowledge the limitations of this preliminary study, including the non-probability sample, and that our findings are not representative of the entire U.S. intersex adult population. Specifically, the age distribution and racial/ethnic composition of our sample differ from national trends; Black/African American respondents were especially underrepresented in this study. There is currently little research on intersex adults with regard to race and ethnicity, and future research should expand on efforts to recruit diverse and representative samples of people with a diagnosed intersex condition. In addition, recruiting participants from support and advocacy groups may produce a cohort that is better informed and connected than average, although we would also expect peer support to be associated with improved mental health outcomes [39]. More than one-third of participants reported never being part of an intersex support group, suggesting that recruitment reached beyond these communities. In addition, we used self-report measures for physical and mental health diagnoses, and our questions did not specify explicitly whether the diagnosis had been given by a healthcare provider. These limitations may introduce bias; however, resource constraints and the fact that many intersex adults are not receiving specialist care limited our ability to confirm self-reported diagnoses through clinical assessment or medical record review. Nonetheless, we designed our survey to maximize robustness, following the CHERRIES guidelines for online surveys (see S4 File) [26], and we mirrored the self-reporting questions used in BRFSS to facilitate comparison. Future research on the physical and health needs of intersex populations including valid screenings and clinical diagnosis of these health conditions is needed. Our survey was also only conducted in English, likely underrepresenting the experiences of limited English speakers. It would be useful to employ longitudinal methods to study health experiences and outcomes over the life course, as well as testing possible public health interventions and investigating the needs and experiences of individuals with different intersex diagnoses.

### Conclusions/public health implications

This is the first national study of the health of intersex adults in the U.S., presenting evidence of poor mental health and functional status compared to the general population, particularly among younger adults aged 18–39. Our findings enhance understanding of the health needs of intersex people and may be relevant for health care professionals who provide care to intersex populations and those leading public health efforts. This study demonstrates the need to expand research and interventions relating to the health of intersex people, particularly targeting mental health and daily function. It is also vital to consider how interventions experienced by intersex infants and children affect health throughout the life course, in order to inform decision-making, promote bodily autonomy, and avoid preventable harms.

## Supporting information

S1 File Additional detail on measures used. (DOCX)

S2 File Survey questions reported on in manuscript. (DOCX)

S3 File Recruitment flyer. (DOCX)

S4 File Checklist for Reporting Results of Internet E-Surveys (CHERRIES). (DOCX)
