# Supplementary material for: A national study on the physical and mental health of intersex adults in the U.S
Source: PLoS One. 2020 Oct 9;15(10):e0240088. doi: 10.1371/journal.pone.0240088 (PMC7546494; doi:10.1371/journal.pone.0240088)
Supplement: S1 File — (DOCX) [file pone.0240088.s001.docx]

**S1 File. Additional detail on measures used.**

*Demographic variables*

Demographic data included age, sex assigned at birth, gender identity, sexual orientation, race/ethnicity, education level (no schooling to professional degree), place of birth (U.S. or elsewhere), annual household income, financial difficulties, employment status, relationship status, health care coverage, and participation in U.S. Armed Services.^1,2^

*Intersex-related health diagnoses*

Questions on diagnoses of intersex variations were based on previous research^3,4^ and expertise of our community partners and research team.

*Physical, psychological, and functional health*

Questions included self-rated physical, functional, and mental health, as well as self-reported health conditions. Health conditions included ever being diagnosed with heart disease, cancers, stroke, osteoporosis, arthritis, and kidney disease. We also asked participants about sensory problems and daily functioning. For mental health, questions included previous diagnosis of depression, anxiety, and post-traumatic stress disorder (PTSD), as well as the 4-item Center for Epidemiological Studies Depression Scale (CES-D), with scores ranging from 0-12 and a cutoff score of ≥4 indicating possible clinical depression.^5,6^

**References**

1. Wallace RB, Herzog AR. Overview of the health measures in the Health and Retirement Study. Journal of Human Resources 1995:S84-S107.

2. Centers for Disease Control and Prevention. Centers for Disease Control and Prevention Behavioral Risk Factor Surveillance System. 2010.

3. Röhle R, Gehrmann K, Szarras-Czapnik M, et al. Participation of adults with disorders/differences of sex development (DSD) in the clinical study dsd-LIFE: design, methodology, recruitment, data quality and study population. BMC Endocrine Disorders 2017;17:52.

4. Tamar-Mattis S, Gamarel KE, Kantor A, Baratz A, Tamar-Mattis A, Operario D. Identifying and Counting Individuals with Differences of Sex Development Conditions in Population Health Research. LGBT Health 2018;5:320-4.

5. Kohout FJ, Berkman LF, Evans DA, Cornoni-Huntley J. Two shorter forms of the CES-D depression symptoms index. Journal of Aging and Health 1993;5:179-93.

6. Melchior LA, Huba G, Brown VB, Reback CJ. A short depression index for women. Educational and Psychological Measurement 1993;53:1117-25.
