# Supplementary material for: A national study on the physical and mental health of intersex adults in the U.S
Source: PLoS One. 2020 Oct 9;15(10):e0240088. doi: 10.1371/journal.pone.0240088 (PMC7546494; doi:10.1371/journal.pone.0240088)
Supplement: S2 File — (DOCX) [file pone.0240088.s002.docx]

**S2 File. Survey questions reported on in manuscript.**

Do you currently live in the United States and U.S. territories (Puerto Rico, Guam, United States Virgin Islands, Northern Mariana Islands, and American Samoa)?

- Yes
- No

Skip To: End of Survey If Do you currently live in the United States and U.S. territories (Puerto Rico, Guam, United States... = No

Some people are assigned male or female at birth but are born with traits including sexual anatomy, reproductive organs, and/or chromosome patterns that may not fit the typical definition of male or female. These traits may be known as variations or differences of sex development (DSD) or intersex.
 
Have you ever been diagnosed by a medical doctor or other health professional with an intersex condition or a ‘Difference of Sex Development (DSD)’ or were you born with (or developed naturally in puberty) genitals, reproductive organs, and/or chromosomal patterns that do not fit standard definitions of male or female?

- Yes
- No

Skip To: End of Survey If Some people are assigned male or female at birth but are born with traits including sexual anatom... = No

Have you ever been told that you had any of the following? (Please check all that apply)

- High blood pressure
- Heart attack (also called a myocardial infarction)
- Angina or coronary heart disease
- Stroke
- Asthma
- Skin cancer
- Other types of cancer
- Chronic Obstructive Pulmonary Disease (COPD), emphesema or chronic bronchitis
- Arthritis, rheumatoid arthritis, gout, lupus or fibromyalgia
- Osteoporosis
- Depressive disorder, including depression, major depression, dysthymia, or minor depression
- Anxiety or an anxiety disorder
- Post-traumatic stress disorder or PTSD
- Kidney disease (not including kidney stones, bladder infection or incontinence)
- Diabetes

Are you deaf or do you have serious difficulty hearing?

- Yes
- No
- Don't know/Not sure
- Prefer not to answer

Are you blind or do you have serious difficulty seeing, even when wearing glasses/contacts?

- Yes
- No
- Don't know/Not sure
- Prefer not to answer

Would you say that in general your health is:

|  | Excellent | Very Good | Good | Fair | Poor | Don't know/Not sure | Prefer not to answer |
| --- | --- | --- | --- | --- | --- | --- | --- |
| Physical Health |  |  |  |  |  |  |  |
| Mental Health |  |  |  |  |  |  |  |

Below is a list of some ways you may have felt or behaved. Please indicate how often you have felt this way **during the past week** by checking the appropriate box for each question.

|  | Rarely or none of the time  (less than 1 day) | Some or a little of the time  (1-2 days) | Occasionally or a moderate amount of the time  (3-4 days) | Most or all of the time  (5-7 days) |
| --- | --- | --- | --- | --- |
| I felt depressed. |  |  |  |  |
| I felt lonely. |  |  |  |  |
| I had crying spells. |  |  |  |  |
| I felt sad. |  |  |  |  |

*This survey asks some questions about previous traumatic experiences including discrimination and violence. Some people may find these questions disturbing, upsetting, or uncomfortable to answer. While we encourage you to answer these questions as honestly as possible, you are welcome to skip any questions that are too upsetting to answer. If you need support, you can call 1-800-273-8255 to talk with someone. Please go to the emergency room or call 911 if you are in crisis and don’t know where to get help.*
 

 Have you ever thought about or attempted to kill yourself?

- Never
- It was just a brief passing thought
- I have had a plan at least once to kill myself but did not try to do it
- I have attempted to kill myself, but did not want to die
- I have attempted to kill myself, and really hoped to die
- Don't know/Not sure
- Prefer not to answer

Because of a physical, mental, or emotional condition, do you have serious difficulty concentrating, remembering, or making decisions?

- Yes
- No
- Don't know/Not sure
- Prefer not to answer

Do you have serious difficulty walking or climbing stairs?

- Yes
- No
- Don't know/Not sure
- Prefer not to answer

Do you have difficulty dressing or bathing?

- Yes
- No
- Don't know/Not sure
- Prefer not to answer

Because of a physical, mental, or emotional condition, do you have difficulty doing errands alone such as visiting a doctor’s office or shopping?

- Yes
- No
- Don't know/Not sure
- Prefer not to answer

Are you Hispanic, Latino/a/x, or of Spanish origin?

- Yes
- No
- Don't know/Not sure
- Prefer not to answer

Were you born in the United States?

- Yes
- No
- Don't know/Not sure
- Prefer not to answer

What is your race? (Check all that apply.)

- American Indian or Alaska Native
- Asian
- Black or African American
- Native Hawaiian or other Pacific Islander
- White
- Another race (please specify)

________________________________________________

- Don't know/Not sure
- Prefer not to answer

How old are you?

________________________________________________________________

What is your current gender identity (Check all that apply.)

- Intersex
- Man
- Transgender man (female-to-male)
- Transgender woman (male-to-female)
- Woman
- Genderqueer
- Non-binary
- Another gender identity (please specify)

________________________________________________

- Don't know/Not sure
- Prefer not to answer

What sex was assigned to you at birth, on your original birth certificate?

- Female
- Male
- Don't know/Not sure
- Prefer not to answer

Please select any of the variations below that you may have been born with or received a diagnosis from a doctor or other healthcare provider (Check all that apply.)

- 5-Alpha reductase deficiency (5-ARD)
- 17-Beta-hydroxysteroid dehydrogenase deficiency
- Aphallia
- Bladder exstrophy
- Clitoromegaly (large clitoris)
- Classic Congenital Adrenal Hyperplasia (Classic CAH)
- Complete Androgen Insensitivity Syndrome (CAIS)
- Cryptorchidism (undescended testicle/s)
- de la Chapelle (XX Male) syndrome
- Epispadias
- Fraser Syndrome
- Gonadal dysgenesis (partial or complete)
- Hypospadias
- Jacobs/ XYY Syndrome
- Kallmann Syndrome
- Klinefelter Syndrome
- Late Onset Congenital Adrenal Hyperplasia (late onset CAH)
- Leydig Cell Hypoplasia
- Micropenis
- Mosaicism involving ‘sex’ chromosomes
- MRKH (Mullerian agenesis; vaginal agenesis; congenital absence of vagina)
- Mullerian (Duct) aplasia
- Ovo-testes (formerly ‘true hermaphroditism’)
- Partial Androgen Insensitivity Syndrome (PAIS)
- Persistent Mullerian Duct Syndrome
- Polycystic Ovary Syndrome (PCOS)/Hyperandrogenism
- Progestin Induced Virilisation
- Swyer Syndrome
- Turner Syndrome (TS, one X chromosome)
- Triple-X Syndrome (XXX)
- XXY/47
- XY/XO Mosaics
- XY-Turner Syndrome
- Another variation (please specify) ________________________________________________
- Unknown

At what age did you find out about your intersex diagnosis?

________________________________________________________________

How would you describe your current sexual orientation? (Check all that apply.)

- Asexual
- Bisexual
- Gay
- Lesbian
- Pansexual
- Queer
- Questioning
- Same-gender loving
- Straight/Heterosexual
- Another (please specify)

________________________________________________

- Don’t know/Not sure
- Prefer not to answer

What is your highest education level completed?

- No schooling
- Grades 1 through 8 (Elementary)
- Grades 9 through 11 (Some high school)
- High school graduate or equivalent (e.g., GED)
- Trade/Technical/Vocational training
- Some college
- 2-year college degree
- 4-year college degree
- Master's degree
- Doctoral degree
- Professional degree (e.g., M.D., J.D., M.B.A.)
- Don’t know/Not sure
- Prefer not to answer

What is your annual household income (before taxes and deductions) from all sources?

- $0-5,000
- $5,001 - 10,000
- $10,001 - 15,000
- $15,001 - 20,000
- $20,001 - 30,000
- $30,001 - 40,000
- $40,001 - 50,000
- $50,001 - 60,000
- $60,001 - 70,000
- $70,001 - 80,000
- $80,001 - 90,000
- $90,001 - 100,000
- $100,001+
- Don’t know/Not sure
- Prefer not to answer

Please tell us about your current employment status. (Check all that apply.) If none of these applies, select "Another."

- Currently working
- Disabled or unable to work
- Homemaker
- Retired
- Unemployed or laid off and looking for work
- Another (please specify)

________________________________________________

- Don’t know/Not sure
- Prefer not to answer

Which of the following best describes your current relationship status?

- Single, never married and never in a civil union/domestic partnership
- Civil union or domestic partnership
- Married
- Separated
- Divorced
- Widowed
- Another (please specify)

________________________________________________

- Prefer not to answer

Are you currently or have you ever been a member of the United States Armed Services?

- Yes
- No
- Don't know/Not sure
- Prefer not to answer

How often do you worry about not meeting your expenses with your current income?

- Never
- Sometimes
- Often
- Always
- Don't know/Not sure
- Prefer not to answer
