# Supplementary material for: A national study on the physical and mental health of intersex adults in the U.S
Source: PLoS One. 2020 Oct 9;15(10):e0240088. doi: 10.1371/journal.pone.0240088 (PMC7546494; doi:10.1371/journal.pone.0240088)
Supplement: S3 File — (DOCX) [file pone.0240088.s003.docx]

**S3 File. Recruitment flyer.**


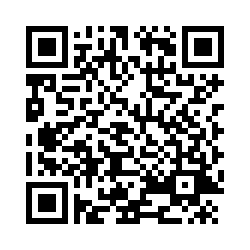

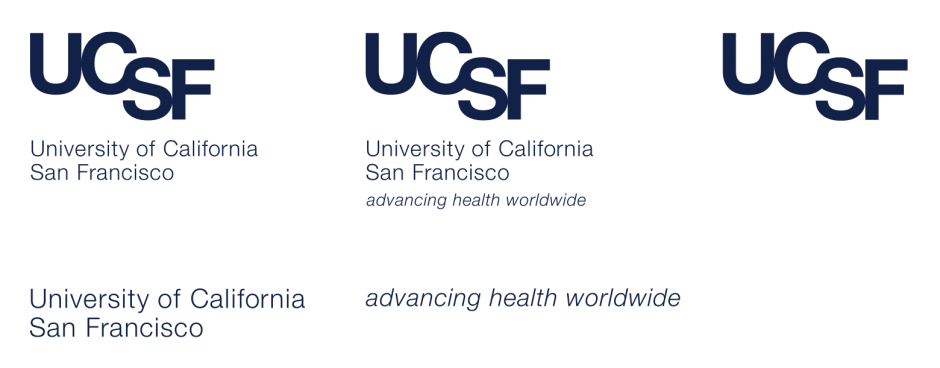
**
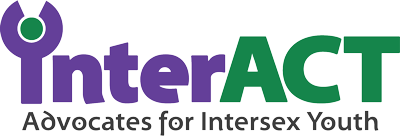
**

Scan QR code with your mobile device

**Participate in a study about the health concerns of the intersex community**

<http://bit.ly/IntersexHealthStudy>

**Enter a raffle for one of 50 $10 Amazon gift cards for completing the survey**

**For more information:**

**Jason Flatt, PhD** **at 415-476-9477**

[**Jason.Flatt@ucsf.edu**](mailto:Jason.Flatt@ucsf.edu)

[**Amy.Rosenwohl-Mack@ucsf.edu**](mailto:Amy.Rosenwohl-Mack@ucsf.edu)
